# Common use of dietary supplements for bipolar disorder: a naturalistic, self-reported study

**DOI:** 10.1186/s40345-015-0029-x

**Published:** 2015-06-02

**Authors:** Michael Bauer, Tasha Glenn, Jörn Conell, Natalie Rasgon, Wendy Marsh, Kemal Sagduyu, Rodrigo Munoz, Ute Lewitzka, Rita Bauer, Maximilian Pilhatsch, Scott Monteith, Peter C. Whybrow

**Affiliations:** Department of Psychiatry and Psychotherapy, Universitätsklinikum Carl Gustav Carus, Technische Universität Dresden, Dresden, Germany; ChronoRecord Association Inc., Fullerton, CA 92834 USA; Department of Psychiatry and Behavioral Sciences, Stanford School of Medicine, Palo Alto, CA 94305-5723 USA; Department of Psychiatry, University of Massachusetts, Worcester, MA 01655 USA; Department of Psychiatry, University of Missouri Kansas City School of Medicine, Stanley, P.O. Box 23430, Kansas City, KS 66283 USA; Department of Psychiatry, University of California San Diego, San Diego, CA USA; Michigan State University College of Human Medicine, Traverse City Campus, Traverse City, MI USA; Department of Psychiatry and Biobehavioral Sciences, Semel Institute for Neuroscience and Human Behavior University of California Los Angeles (UCLA), Los Angeles, CA 90095 USA

## Abstract

**Background:**

Dietary supplements are taken by about half of Americans. Knowledge of dietary supplement use is important because they may interact with prescription drugs or other supplements, cause adverse reactions including psychiatric symptoms, or contain inherently toxic ingredients or contaminants. This study explores the use of dietary supplements by patients with bipolar disorder in the US.

**Methods:**

Data were obtained from an ongoing, naturalistic study of patients with bipolar disorder who received pharmacological treatment as usual. The patients self-reported their daily mood, sleep, and medications taken, including all drugs prescribed for bipolar disorder or that the patient felt impacted their mood. These included other prescribed drugs, over-the-counter drugs and dietary supplements. Drugs that received premarketing approval from the FDA were not included as dietary supplements. Patient demographics and daily medication use were characterized.

**Results:**

Data were available from 348 patients in the US who returned a mean 249.5 days of data. In addition to prescribed psychiatric drugs, 101 of the 348 patients (29 %) used a dietary supplement for at least 7 days and 69 (20 %) used a supplement long term (for at least 50 % of days). Of the 101 supplement users, 72 (71.3 %) took one supplement daily. The 101 patients tried over 40 different supplements, and the long-term users took 19 different supplements. The most commonly taken supplements for both groups were fish oil, B vitamins, melatonin, and multivitamins. Patients using supplements were more likely to be white (*p* < 0.001), older (*p* = 0.009), and ill for more years (*p* = 0.025).

**Conclusions:**

Many patients with bipolar disorder use dietary supplements in addition to prescribed drugs. Physicians should obtain detailed information about all dietary supplements taken by patients with bipolar disorder.

## Background

Dietary supplements, including vitamins, minerals, herbals and botanicals, amino acids, enzymes, and other products are used by about half the US population (Gahche et al. 2011). Sales of dietary supplements generated about US$32 billion in revenue in 2012 (Lariviere 2012; NIH 2011) or about one tenth of that generated by prescription drugs (Bartholow 2013). In 2008, the FDA estimated there were 55,600 distinct dietary supplement product labels manufactured by 1460 firms (Federal Register 2009). The most popular supplements are multivitamin/mineral products, taken by about one third of Americans (Bailey et al. 2011; NIH 2013). About three fourths of dietary supplements used by the general public are selected by personal choice rather than prescribed by a physician (Bailey et al. 2013). Less is known about the use of dietary supplements by people with mental illness, with studies reporting that 15–36 % take herbal and/or dietary supplements (Elkins et al. 2005; Knaudt et al. 1999; Matthews et al. 2003; Russinova et al. 2002; Wu et al. 2007).

Physician knowledge of patient use of dietary supplements is important because of the potential for interactions with prescription drugs or other supplements (Izzo and Ernst 2009), potential adverse reactions including psychiatric (Ernst 2003; Tsai et al. 2012), potential dangers to special populations such as pregnant women, and the inherent toxicity of some ingredients (Kroll 2004). Only about one third of patients, with and without mental illness, disclose the use of dietary supplements to their physician (Blendon et al. 2013; Elkins et al. 2005, Gardiner et al. 2006; Keaton et al. 2009; Kennedy 2005; Matthews et al. 2003; McCrea and Pritchard 2011; Mehta et al. 2008). Furthermore, the use of dietary supplements may not be documented in a patient’s electronic medical record (Staroselsky et al. 2008). This information may be particularly important for patients with bipolar disorder since most are prescribed polypharmacy (Bauer et al. 2013b; Lin et al. 2006), and the risk of drug interactions increases with the number of drugs taken (Goldberg et al. 1996; Köhler et al. 2000). This study explores the use of dietary supplements by patients with bipolar disorder who received pharmacologic treatment as usual, based on daily self-reported data.

## Methods

All data were obtained from an ongoing, naturalistic, international study in which patients with bipolar disorder self-reported daily their mood, sleep, and medications taken (Bauer et al. 2009; Bauer et al. 2013a,b; Bauer et al. 2013c). Study participants must have a diagnosis of bipolar disorder by the DSM-IV criteria, be at least 18 years old, receive pharmacological treatment as usual, and be willing to use ChronoRecord self-reporting software daily on a home computer. The study has minimal inclusion criteria to better represent the heterogeneity of routine clinical practice. The diagnosis of bipolar disorder was made by the prescribing psychiatrist in a clinical interview, and all patients received treatment as usual. All participants were volunteers, primarily recruited by the prescribing psychiatrist, and were informed about the study prior to providing written informed consent. The study was approved by the Ethical Committee of the Medical Faculty of the Technische Universität Dresden, and is in compliance with the Helsinki Declaration. Patents received about a half hour of training, in person or by telephone, before entering data.

The ChronoRecord database includes patients who reside in several countries. However, because of international differences in the definition and regulation of dietary supplements (Getman 2015), only data from patients who reside in the US were included in this analysis.

### Data collection

The patients entered mood, sleep, medications, and significant life events daily and weight weekly into the ChronoRecord software. The daily self-ratings for mood in ChronoRecord were previously validated with clinician ratings on the Hamilton Depression Rating Scale (HAMD) and the Young Mania Rating Scale (YMRS) (Bauer et al., 2004, 2008). For rating mood, ChronoRecord uses a 100-unit visual analog scale between the extremes of mania and depression. Based upon the validation studies (Bauer et al., 2004, 2008), a mood entry less than 40 was considered depression, 40–60 euthymia, and greater than 60 hypomania/mania. The depression ratings varied from mild (entry of 20–39) to moderate-severe (entry of 0–19), and the mania ratings varied from hypomania (entry of 61–80) to moderate-severe (entry of 81–100).

During patient training, a medication list was created for each patient. The medication list includes all drugs prescribed for bipolar disorder and any other medications that the patient felt impacted their mood, including prescribed medications, over-the-counter (OTC) drugs, and dietary supplements. The prescribed psychotropic drugs were selected from a list in the software, displayed by brand and generic names, including some well-known supplements such as St. John’s wort. The patient could add a drug or dietary supplement not included in the software list and could modify the list of drugs taken throughout the study period as needed. For each selected drug, the pill strength was chosen from a list of available strengths. Every day, for each drug, the patient entered the total number of pills taken. Patients could enter partial pills (1/4, 1/2, or 3/4) for tablets but not capsules. If a drug was not taken, the patient entered 0 pills for that drug for that day. Data not entered on one day could be entered at a later date. The software includes many error checking steps such as to prevent entry for a future date.

### Dietary supplements

The use of a dietary supplement was defined as taking one supplement for at least 7 consecutive or non-consecutive days during the study. Long-term use of a dietary supplement was defined as taking one supplement for at least 50 % of the study days. Drugs taken in this study that were approved by the FDA, such as the prescription drug Lovaza (omega-3-acid ethyl esters) or OTC drug Unisom (doxylamine), were not included as dietary supplements. The specific formulations or manufacturer of the dietary supplements were not obtained.

### Statistical analysis

A minimum of 180 days of patient data was required for inclusion in the analysis. The characteristics of patients using and not using dietary supplements were compared. Distributions of categorical variables were compared using the Pearson’s two-sided asymptotic chi-squared test. Mean values of continuous variables were compared using the independent sample two-sided *t* test. Unequal variance was assumed for all *t* tests. A *p* value of less than 0.05 was considered statistically significant. SPSS version 22.0 was used for all calculations.

## Results

Three hundred forty-eight patients in the US returned a mean of 249.5 days of data. Of the 348 patients, 101 (29 %) used a dietary supplement and 69 (20 %) were long-term users. The 101 users took a dietary supplement for a mean of 66.5 % of the days (SD 33.6), while the 69 long-term users took a dietary supplement for a mean of 86.7 % of days (SD 14.9). The demographics of the patients using and not using dietary supplements are shown in Table 1. The patients using a dietary supplement were older, white, and had more years of illness. These conclusions did not change for the long-term users. The patients using dietary supplements tried 40 different supplements, summarized in Table 2, with long-term users taking 19 different supplements. Of the supplement users (*n* = 101), the most commonly used supplements were fish oil (*n* = 62), B vitamins (*n* = 25), melatonin (*n* = 20), and multivitamins (*n* = 10). The same supplements were most commonly used by the 69 long-term users. Of the 101 supplement users, 72 (71.3 %) were taking one supplement, 9 (8.9 %) were taking two supplements, 20 (19.8 %) were taking three or more supplements (10 taking three supplements and 10 taking four or more). Of the 69 long-term users, 58 (84 %) were taking one supplement, 6 (8.7 %) were taking two supplements, and 5 (7.2 %) were taking three or more supplements (4 taking three supplements and 1 taking five supplements).

**Table 1:** Patient demographics by using or not using dietary supplements (*N* = 348)

	Using (*N* = 101)	Not using (*N* = 247)	Test	Value	*df*	*P*
Gender *N* (%)			*Χ* ^2a^	1.224	1	0.269
Male	29 (28.7)	57 (23.1)				
Female	72 (71.3)	190 (76.9)				
Diagnosis *N* (%)			*Χ* ^2^	2.489	2	0.288
Bipolar I	51 (51.0)	146 (60.0)				
Bipolar II	41 (41.0)	83 (34.2)				
NOS	8 (8.0)	14 (5.8)				
Employment *N* (%)			*Χ* ^2^	0.831	2	0.660
Working full-time	49 (49.5)	112 (46.5)				
Disabled	22 (22.2)	65 (27.0)				
Others	28 (28.3)	64 (26.5)				
Education *N* (%)			*Χ* ^2^	1.643	2	0.440
High school	7 (6.9)	27 (11.3)				
Some college	30 (29.7)	73 (30.4)				
College graduate	64 (63.4)	140 (58.3)				
Marital status *N* (%)			*Χ* ^2^	0.412	2	0.814
Married	49 (50.0)	114 (48.3)				
Single	34 (34.7)	90 (38.1)				
Divorced	15 (15.3)	32 (13.6)				
Ethnicity *N* (%)			*Χ* ^2^	14.875	1	<0.001
White	87 (86.1)	162 (65.6)				
Non-white	14 (13.9)	85 (34.4)				
Age mean age (SD)	41.8 (11.1)	38.4 (10.7)	*t* ^b^	−2.627	180	0.009

^a^Pearson’s chi-square, asymptotic two-sided

^b^Equal variances not assumed

**Table 2:** Dietary supplements taken by supplement users (*N* = 101)

Type	Supplement
Vitamins	
	Multivitamins
	Vitamin B (folate, B_6_, B_12_, niacin, and combination products)
	Vitamin C
	Vitamin D
	Vitamin E
Minerals	
	Calcium
	Chromium
	Iron
	Magnesium
	Zinc
Herbs and nutritional products	
	Artemisinin
	Caffeine
	DHEA
	Echinacea
	Fish oil
	GABA
	Garlic
	Ginkgo
	Glucosamine (with/without chondrotin)
	Karaela
	Melatonin
	Methylsulfonylmethane (MSM)
	Primrose oil
	Probiotics
	S-adenosylmethionine
	St. John’s wort
	Thyroid support products
	Tryptophan
	Tyrosine
	Valerian
	Weight loss products

All the patients taking a supplement were also taking prescribed medications for bipolar disorder. As shown in Table 3, there was no difference in the use of mood stabilizers, antidepressants, antipsychotics, or benzodiazepines between those using and not using supplements. There was also no difference in reported symptoms of mania or depression, including severe symptoms, between those using and not using supplements. These conclusions for medication use or mood reporting did not change for the long-term users. Of the 69 long-term supplement users, 51 were taking both a mood stabilizer and a supplement. These 51 patients skipped taking mood stabilizers on 5.2 % of the days and skipped taking supplements on 14.3 % of the days.

**Table 3:** Clinical characteristics by using or not using dietary supplements (*N* = 348)

Clinical characteristics	Using dietary supplements (*N* = 101)	Not using dietary supplements (*N* = 247)	Test	Value	*df*	*P*
Taking mood stabilizer *N* (%)^a^			*Χ* ^2b^	1.367	1	0.242
Yes	73 (72.3)	193 (78.1)				
No	28 (27.7)	54 (21.9)				
Taking antidepressant *N* (%)^a^			*Χ* ^2^	0.573	1	0.449
Yes	54 (53.5)	143 (57.9)				
No	47 (46.5)	104 (42.1)				
Taking antipsychotic *N* (%)^a^			*Χ* ^2^	0.340	1	0.560
Yes	41 (40.6)	92 (37.2)				
No	60 (59.4)	155 (62.8)				
Taking benzodiazepine *N* (%)^a^			*Χ* ^2^	0.016	1	0.901
Yes	26 (25.7)	62 (25.1)				
No	75 (74.3)	185 (74.9)				
Percentage of days depressed, mean (SD)	22.4 (22.4)	22.2 (23.6)	*t* ^c^	−0.074	194	0.941
Percentage of days euthymic, mean (SD)	70.5 (24.3)	68.7 (26.8)	*t*	−0.584	204	0.560
Percentage of days manic, mean (SD)	7.1 (10.3)	9.0 (13.5)	*t*	1.437	241	0.152
Percentage of days severely depressed, mean (SD)	8.3 (15.1)	7.4 (15.7)	*t*	−0.484	192	0.629
Percentage of days severely manic, mean (SD)	0.7 (2.4)	1.2 (4.7)	*t*	1.380	330	0.169
Hospitalizations, mean number (SD)	2.1 (4.7)	1.9 (3.2)	*t*	−0.482	141	0.630
Age of onset, mean age (SD)	20.0 (8.2)	20.6 (9.7)	*t*	0.545	198	0.586
Years of illness, mean years (SD)	21.9 (13.1)	18.45 (11.9)	*t*	−2.262	174	0.025

^a^Taking for 50 % or more days in study

^b^Pearson’s chi-square, asymptotic two-sided

^c^Equal variances not assumed

## Discussion

Thirty percent of patients with bipolar disorder in this study used dietary supplements in addition to prescribed medications, with one in five patients using supplements for the long term. Since this study did not include dietary supplements used for other diseases or general health, the results are likely to underestimate the total number of patients using dietary supplements and the total supplements taken. Nevertheless, these findings remain consistent with prior research. About 13–52 % of adults in the general population use dietary supplements and prescription medications concurrently (Farina et al. 2014; Gardiner et al. 2006; McCrea and Pritchard 2011; Qato et al. 2008), increasing when including vitamins and minerals. The finding that dietary supplement use was highest among older, white individuals was also consistent with prior research (Bailey et al. 2011; Gahche et al. 2011; Schaffer et al. 2003), although some studies also report higher use in women than men (Gahche et al. 2011; Gardiner et al. 2006; Schaffer et al. 2003).

In this study, patients using both dietary supplements and mood stabilizers skipped taking the supplements nearly three times more often than the mood stabilizers. Similarly, in prior research, complementary and alternative medicines did not decrease adherence to medications prescribed for psychiatric disorders (Ennis 2014) or general medical disorders (Cherniack 2011). The long-term use of supplements had no impact on self-reported mood symptoms, although individual supplements were not analyzed. While the efficacy of dietary supplements in the treatment of bipolar disorder is outside the scope of this analysis, most review articles do not recommend routine use of dietary supplements yet recognize that some products have potential adjunctive benefit (Andreescu et al. 2008; Rakofsky and Dunlop 2014; Sagduyu et al. 2005; Sarris et al. 2011). However, adverse effects of some supplements may destabilize mood (Ernst 2003; Werneke et al. 2006). For example, St. John’s wort, S-adenosylmethionine, and ginseng may induce mania (Andreescu et al. 2008; Norelli and Xu 2014), supplements containing serotonergic agents and stimulants may induce anxiety (McCarthy et al. 2014), and melatonin may increase daytime sleepiness (Werneke et al. 2006).

The findings of this study highlight the need to ask all patients with bipolar disorder about their use of dietary supplements. While the vast majority of dietary supplements are taken safely by millions daily, many issues remain relating to the formulations of commercial dietary supplements. Analytical testing of many commonly taken dietary supplement products has found a wide deviation between the ingredient amount stated on the label and the measured amount (Lockwood 2011). For example, testing of products purchased in US retail outlets have reported potency between 62–184 % for fish oil EPA and DHA (Kleiner et al. 2014) and between 9–146 % for vitamin D (cholecalciferol) (LeBlanc et al. 2013). A recent investigation of top-selling store brands of herbal supplements at four national retailers found that 80 % of the products did not contain the herbs stated on their labels (O’Connor 2015). Differences in the purity and integrity of melatonin products may contribute to the observed variations in effective doses and serum concentrations (de Rooij et al. 2014). Other quality concerns with dietary supplements include adulterants not declared on the labels such as prescription drugs, prohibited stimulants, prohormones, anabolic steroids, chemical toxins or other chemical ingredients (Geyer et al. 2008; Vaclavik et al. 2014), and microbial contamination (Tournas 2009). Clinically relevant amounts of T3 and/or T4 were present in nine out of ten dietary or “thyroid support” supplements purchased online (Kang et al. 2013). The supplements most frequently adulterated are those advertised for the support of chronic illnesses, obesity, and for athletes (Vaclavik et al. 2014).

Unlike drugs with a single active ingredient, supplements are often complex mixtures of ingredients, often of biological origin, and have multiple pharmacological properties (Foster et al. 2005). As reviewed in detail elsewhere, many dietary supplements used by patients with bipolar disorder have the potential for pharmacokinetic and pharmacodynamic interactions with prescribed drugs or other supplements (Izzo and Ernst 2009; Gardiner et al. 2008). Pharmacokinetic interactions occur when one drug impacts the absorption, distribution, metabolism, or excretion of another drug, causing the blood level to increase or decrease (Sandson et al. 2005). For example, St. John’s wort may reduce effectiveness of benzodiazepines and methadone due to induction of cytochrome P450 3A4 enzyme (Izzo and Ernst 2009). Pharmacodynamic interactions occur when drugs act upon the same or related receptors, causing additive or oppositional effects (Sandson et al. 2005). For example, St. John’s wort may interact with serotonergic agents such as SSRI antidepressants to produce serotonin syndrome (Izzo and Ernst 2009; Gardiner et al. 2008). In a literature review of 1491 drug-dietary supplement interactions, prescription drugs that affect the central nervous system or cardiovascular system were involved in the most interactions with dietary supplements, and the most frequently involved supplements were products containing St. John’s wort, magnesium, calcium, iron, and ginkgo (Tsai et al. 2012). Adverse events with dietary supplements are underreported for several reasons including the expectation that consumers will recognize and report these to the FDA (GAO 2013). Furthermore, many dietary supplements may not be included in software that checks for drug interactions. The use of herbal products may also result in abnormal laboratory test results (Dasgupta 2003).

Given their frequent use in bipolar disorder, it is also important to recognize that the general public has many misconceptions about dietary supplements. Many incorrectly assume that the FDA evaluates dietary supplement products for safety and efficacy and approves product advertisements (Ashar et al. 2008; Dodge et al. 2011; Keaton et al. 2009; Pillitteri et al. 2008). Dietary supplements and products labeled “natural” are often perceived as being safer than OTC or prescription drugs (Dennehy et al. 2005; Nichter and Thompson 2006). In a survey of the general public (*n* = 1027), 29 % felt it was not necessary to follow the recommended dosages for dietary supplements, as compared with only 9 % for prescription drugs (Gibson and Taylor 2005). Additionally, warning labels on dietary supplement products intended to decrease consumer perceptions of safety may have unintended consequences and increase some consumer perceptions of efficacy (Mason et al. 2007). Only one fourth of users would stop using a supplement if the regulators said it is ineffective (Blendon et al. 2013).

It is also difficult for patients to find high-quality information about dietary supplements. Many pharmacists do not have sufficient knowledge of dietary supplements to provide counselling (Boon et al. 2009). Dietary supplements are often purchased on the Internet, and many of these web sites provide inadequate information to the consumer (Monteith et al. 2013). In a survey of 1179 web sites for 13 common herbal products, only 8 % of retail sites contained any safety information, and only 10.5 % recommended consultation with a health-care professional (Owens et al. 2014).

There are limitations to this study. All data in the study were self-reported, and daily access to a home computer was required. The study included more females than males and did not include patients with bipolar disorder who were not receiving treatment. However, the demographic profile of the patients who use ChronoRecord is similar to that in other studies of bipolar disorder (Bauer et al. 2009; Bauer et al. 2012). Patient reluctance to report dietary supplement use may also contribute to underestimation of usage. We assume that most physicians do not routinely ask patients about supplement use. It is not known if use of the dietary supplement in this study was initiated by the patient or recommended by the physician. This database does not include medications taken for diseases other than bipolar disorder, so the potential for interactions with dietary supplements could not be accurately determined. Only US data were included in this analysis, although dietary supplements are commonly used worldwide. There are not enough study participants to analyze for ethnic differences in the use of dietary supplements, which have been noted in prior research (Albright et al. 2012).

## Conclusions

Patients with bipolar disorder commonly take dietary supplements in addition to prescription medications. About three out of ten patients used dietary supplements, with two in ten of these taking supplements for the long-term. The patients tried about 40 different supplements, with fish oil, vitamin B preparations, multivitamins, and melatonin taken most frequently. Dietary supplements have the potential to interact with prescription drugs and other supplements and to cause adverse reactions. Many quality issues remain with formulations of commercial dietary supplements. The physician should obtain detailed information about all dietary supplements taken by patients with bipolar disorder.
